# A prospective study to examine the epidemiology of methicillin-resistant *Staphylococcus aureus* and *Clostridium difficile* contamination in the general environment of three community hospitals in southern Ontario, Canada

**DOI:** 10.1186/1471-2334-12-290

**Published:** 2012-11-08

**Authors:** Meredith C Faires, David L Pearl, William A Ciccotelli, Karen Straus, Giovanna Zinken, Olaf Berke, Richard J Reid-Smith, J Scott Weese

**Affiliations:** 1 Department of Population Medicine, University of Guelph, Guelph, ON, Canada; 2 Infection Prevention and Control, Grand River Hospital, Kitchener, ON, Canada; 3 Department of Pathology and Molecular Medicine, McMaster University, Hamilton, ON, Canada; 4 Infection Prevention and Control, St. Mary’s General Hospital, Kitchener, ON, Canada; 5 Department of Mathematics and Statistics, University of Guelph, Guelph, ON, Canada; 6 Department of Pathobiology, University of Guelph, Guelph, ON, Canada

**Keywords:** Methicillin-resistant *Staphylococcus aureus*, *Clostridium difficile*, Hospital, General environment, Risk factors, Livestock-associated MRSA strain, *Spa* typing, Ribotyping

## Abstract

**Background:**

The hospital environment has been suggested as playing an important role in the transmission of hospital-associated (HA) pathogens. However, studies investigating the contamination of the hospital environment with methicillin-resistant *Staphylococcus aureus* (MRSA) or *Clostridium difficile* have generally focused on point prevalence studies of only a single pathogen. Research evaluating the roles of these two pathogens, concurrently, in the general hospital environment has not been conducted. The objectives of this study were to determine the prevalence and identify risk factors associated with MRSA and *C. difficile* contamination in the general environment of three community hospitals, prospectively.

**Methods:**

Sampling of environmental surfaces distributed over the medicine and surgical wards at each hospital was conducted once a week for four consecutive weeks. Sterile electrostatic cloths were used for environmental sampling and information regarding the surface sampled was recorded. For MRSA, air sampling was also conducted. Enrichment culture was performed and *spa* typing was performed for all MRSA isolates. For *C. difficile*, isolates were characterized by ribotyping and investigated for the presence of toxin genes by PCR. Using logistic regression, the following risk factors were examined for MRSA or *C. difficile* contamination: type of surface sampled, surface material, surface location, and the presence/absence of the other HA pathogen under investigation.

**Results:**

Overall, 11.8% (n=612) and 2.4% (n=552) of surfaces were positive for MRSA and *C. difficile*, respectively. Based on molecular typing, five different MRSA strains and eight different *C. difficile* ribotypes, including ribotypes 027 (15.4%) and 078 (7.7%), were identified in the hospital environment. Results from the logistic regression model indicate that compared to computer keyboards, the following surfaces had increased odds of being contaminated with MRSA: chair backs, hand rails, isolation carts, and sofas.

**Conclusions:**

MRSA and *C. difficile* were identified from a variety of surfaces in the general hospital environment.

Several surfaces had an increased risk of being contaminated with MRSA but further studies regarding contact rates, type of surface material, and the populations using these surfaces are warranted.

## Background

Methicillin-resistant *Staphylococcus aureus* (MRSA) and *Clostridium difficile* are two leading hospital-associated (HA) pathogens with both being important causes of patient morbidity and mortality [1,2], outbreaks [3,4], and substantial healthcare costs [5,6]. While data are incomplete and sometimes conflicting, the environment has been suggested as being an important source for the transmission of HA pathogens [7-9], however, the precise role of environmental contamination in HA infections remains unclear [10]. Several studies have been conducted concerning the epidemiology of MRSA [8,11,12] and *C. difficile*[13-16] transmission within healthcare facilities. Although studies have been published surveying the environment in patient rooms [12,15,17-22], information pertaining to MRSA and *C. difficile* contamination in the general hospital environment is limited. Furthermore, there has been little research involving community hospitals, since epidemiological and microbiological studies have focused almost exclusively on tertiary healthcare facilities [11,16-18]. While this approach provides important information, it is unclear whether results from tertiary care facilities are applicable to smaller community hospitals, and therefore whether recommendations based on tertiary care hospital data are broadly applicable.

Studies investigating contamination of the hospital environment with MRSA or *C. difficile* have generally focused on one pathogen [11-15]. Research evaluating the concurrent roles of these two HA pathogens in the general hospital environment has not been conducted and potential associations or commonalities between environmental contamination with MRSA and *C. difficile* have not been investigated. As some hospitals lack the laboratory equipment necessary to culture *C. difficile,* the investigators hypothesized that if there was a correlation or association between MRSA and *C. difficile* at the sample level, consequently, hospitals that conducted environmental audits or surveillance for MRSA could reasonably presume that the surface was also contaminated with *C. difficile.* Furthermore, longitudinal information pertaining to risk factors for environmental contamination, such as specific environmental surfaces sampled or surface material (e.g., fabric versus plastic as it relates to decontamination potential) outside of an outbreak scenario has not been explored. The data provided by this type of research can be used by infection control personnel to guide surveillance, and assess and implement environmental control measures for reducing contamination of the general hospital environment with MRSA and *C. difficile*.

The objectives of this study were to determine the prevalence of MRSA and *C. difficile* in the general hospital environment, determine what environmental surfaces and type of surfaces were most likely contaminated with MRSA and *C. difficile*, identify if access by staff or the public was associated with increased MRSA and *C. difficile* contamination, and compare MRSA and *C. difficile* strains between patients and the environment.

## Methods

### Setting

Three community hospitals, referred to as A, B, and C, located in southern Ontario, Canada, participated in this study. Information pertaining to each healthcare facility is presented in Table 1. Environmental sampling was conducted in February and March, 2010 in hospitals A and B and June 2010 for hospital C. During the study period, none of the hospitals identified MRSA or *C. difficile* outbreaks. This study was approved by the research ethics boards of the University of Guelph and the participating hospitals.

**Table 1:** Description of participating hospitals

**Features**	**Hospital**		
	**A**	**B**	**C**
Number of beds	345	191	226
Number of in- and out- patient visits, 2010	>200,000	>150,000	>100,000
Range of MRSA infection^1^ rates per 1,000 patient-days, 2009 and 2010	2009: 0.00 – 0.08	2009: 0.00 – 1.07	2009: 0.00 – 0.17
	2010: 0.00 – 0.11	2010: 0.00 – 0.08	2010: 0.00 – 0.08
MRSA infection^1^ rates per 1,000 patient-days, 2010 for month of sampling	0.07	0.08	0.00
Range of *Clostridium difficile* infection rates per 1,000 patient-days, 2009 and 2010	2009: 0.13 – 0.67	2009: 0.24 – 1.31	2009: 0.00 – 0.56
	2010: 0.00 – 0.36	2010: 0.00 – 0.98	2010: 0.00 – 0.79
*C. difficile* infection rates per 1,000 patient-days, 2010 for month of sampling	0.26	0.76	0.79

^1^ Rates for bacteremia.

### Sampling

Structured sampling of a pre-determined set of environmental surfaces distributed over the general medicine and surgical wards at each participating hospital was conducted one day a week for four consecutive weeks. The selection of surfaces to be investigated for contamination was based on data presented in previous studies [11,13,20] and through discussion with hospital staff regarding cleaning and disinfection practices. Environmental surfaces were sampled four times as this would provide good quality data regarding contamination in the general ward environment. Surfaces sampled were broadly replicable between wards and hospitals, in addition to sites that were relevant and specific to each ward. Surfaces were sampled prior to that day’s cleaning by housekeeping staff. All hospitals used a one step cleaner and disinfectant protocol and wards were cleaned once per day. No changes in the cleaning protocol were instituted during the study period.

Dry sterile electrostatic cloths (Swiffer™, Proctor and Gamble, USA) were used for environmental sampling. Using a gloved hand, the investigator wiped the electrostatic cloth over half the environmental surface to be sampled, up to a maximum of 20 cm x 20 cm. The cloth was then placed in a sterile collection bag (Whirl-Pak®, Nasco, California, USA). A second electrostatic cloth was used to sample the other half of the environmental surface. Gloves were changed between each sample. During each hospital visit, two electrostatic cloths were not used for sampling but were handled and processed in parallel, to act as negative controls for quality assurance. Information collected with each surface sampled included: date, hospital, hospital ward, type of surface (e.g., sofa), surface material (e.g., fabric), and if the surface was accessible by the public or only by hospital staff. For MRSA, impact air sampling was also performed using an air IDEAL ®3P air sampler (Biomérieux, Saint Laurent, Quebec, Canada) in elevator areas, nurses’ stations, and waiting rooms. A total of 500 L of air was collected onto selective MRSA culture agar (BBL CHROMagar MRSA, Becton, Dickinson and Company, Sparks, Maryland, USA) over a five minute period. Air sampling was conducted at a height of 1 metre and a minimum of 1 metre from the nearest surface.

From all participating facilities, MRSA isolates from patients hospitalized in the medical and surgical wards during the study period were obtained from the microbiology laboratory following MRSA confirmation. Isolates were collected from culture plates using a culture swab with Stuart’s media. Stool samples from patients diagnosed with a *C. difficile* infection by a fecal toxin test (Tox A/B Quik Chek, TechLab, Blacksburg, Virginia, USA) were obtained from Hospital C only. All isolates were collected at the discretion of medical personnel. For MRSA and *C. difficile*, only one isolate per patient was collected.

### Processing

For MRSA, cloths were immersed in 30 ml of enrichment broth containing tryptone (10g/L), sodium chloride (75 g/L), mannitol (10g/L), and yeast extract (2.5g/L) and incubated at 35°C, aerobically, for 24 hours. Following incubation, approximately 5 μL of broth was inoculated onto MRSA Chromogenic agar (BBL CHROMagar MRSA, Becton, Dickinson and Company, Sparks, Maryland, USA) and incubated, aerobically, at 35°C for 24-48 hours. Colonies with an appearance consistent with MRSA were sub-cultured onto blood agar (Oxoid, Nepean, Ontario, Canada) and identified as *S. aureus* by Gram stain, catalase test, tube coagulase test, and *S. aureus* latex agglutination assay (Pastorex Staph-plus, Bio-Rad Laboratories Ltd, Mississauga, Ontario, Canada). The presence of methicillin-resistance was confirmed by testing for penicillin-binding protein 2a (MRSA latex agglutination test, Oxoid Ltd., Hants, UK). For air samples, agar plates were incubated and processed as described above. For patient isolates, culture swabs were streaked onto blood agar (Oxoid, Nepean, Ontario, Canada) and processed as described above. Molecular typing of MRSA was conducted using sequence analysis of the X region of the staphylococcal protein A gene (*spa* typing) [23]. Sequences were then analyzed using two different methods; eGenomics software [24] and the Ridom system [25]. Based on eGenomics, *spa* types are reported using a numerical system (e.g., *spa* type 2) whereas Ridom *spa* types are reported using a numerical system preceded by a ‘t’ (e.g., t002). The *spa* types obtained were compared to epidemic MRSA clones that are frequently found in North America [26]. All MRSA isolates were investigated for the *luk*F-PV gene encoding the Panton-Valentine leukocidin toxin by real-time PCR [27].

For *C. difficile*, cloths were immersed in 30 ml of brain-heart infusion broth supplemented with 0.1% sodium taurocholate and incubated anaerobically at 37°C for 5 days. A 2 ml aliquot of broth was alcohol shocked by addition of an equal volume of anhydrous alcohol and incubated at room temperature for one hour followed by centrifugation at 4,000 rpm for 10 min. The resulting pellet was then inoculated onto *C. difficile* moxalactam-norfloxacin agar (Oxoid, Nepean, Ontario, Canada) and incubated anaerobically for 24-96 hours at 37°C. Presumptive colonies were sub-cultured onto blood agar (Oxoid, Nepean, Ontario, Canada) and identified as *C. difficile* based on characteristic colony morphology, odour, and production of L-proline-aminopeptidase (Prodisk, Remel, Lenexa, Kansas, USA). For patient isolates, approximately 1 g of feces was inoculated into 9 ml of brain-heart infusion broth and processed as described above. All isolates identified as *C. difficile* were investigated for the presence of genes for toxin A (*tcdA*) [28], toxin B (*tcdB*) [29], and binary toxin (*cdtA*) [30] using PCR. Ribotyping was also performed [31]. When a ribotype pattern was known to be an international ribotype based on comparison to reference strains, the appropriate numerical designation (e.g., 027) was assigned. Otherwise, an internal laboratory designation was assigned. Toxinotyping [32] was performed on a representative of each toxigenic ribotype.

### Statistical analysis

#### Descriptive statistics

The prevalence of MRSA and *C. difficile* contamination by visit, ward, surface material, surface location, and type of surface was determined for each hospital. If the overall prevalence of MRSA or *C. difficile* in the general ward environment was at least 10%, a regression model was constructed to identify risk factors for contamination.

### Statistical models

Initially, a multilevel logistic regression model was constructed due to the hierarchical structure of the data. The four-level hierarchical structure for this analysis consisted of repeated samples nested in surfaces that were nested in wards that were nested in hospitals. For model building, the dependent variables were the presence or absence of MRSA or *C. difficile* on a surface. Independent variables investigated included surface material, surface location, type of surface sampled, and the presence/absence of the other HA pathogen under investigation. To control for clustering, the multilevel logistic regression model included a fixed effect for hospital and random intercepts for ward and surface.

The Spearman’s rank correlation test was used to identify correlations between independent variables and the correlation between MRSA and *C. difficile* contamination at the sample level. Independent variables with a correlation >0.8 were investigated and only the variable that was more biologically plausible was included in the model to avoid issues associated with collinearity [33]. Additionally, for common surfaces, a paired exact logistic regression was conducted to determine if there were any significant differences in the probability of a surface being contaminated with MRSA or *C. difficile*.

Univariable logistic regression models were constructed to screen the independent variables with each dependent variable using a significance level of α ≤0.25. Multivariable models were constructed by a manual backwards step-wise procedure starting with all significant variables based on the liberal P-value. Confounding was evaluated by examining the effect of the removed variables on the coefficients of the remaining variables. A variable was deemed to be a confounder if it was not an intervening variable and the log odds of a statistically significant independent variable changed by at least 20% [34]. Interaction terms were examined for all independent variables in the final main effects model. Using the final multivariable model, the investigators examined contrasts for independent variables with >2 categories. These contrasts allowed the researchers to investigate significant differences between any two categories.

Random intercepts were removed from the model if, based on a likelihood ratio (LR) test, they were not statistically significant and they did not confound the observed associations. Similarly, Akaike’s Information Criteria (AIC) were examined among models with and without the random intercepts to assess which model provided the best fit. If random effects were included in the final model, standardized Pearson residuals were assessed to identify outliers. In addition, normality and homogeneity of variance for the best linear unbiased predictors were examined to assess model fit. If random effects were not included in the final model, a Pearson χ^2^ test was used to assess model fit and standardized Pearson residuals were evaluated to identify outliers.

All descriptive statistics, model building, and analyses were performed using Stata 11.0 (StataCorp LP, College Station, Texas, USA). All tests were two-sided and statistical significance was based on an α ≤ 0.05.

## Results

### Descriptive statistics

Due to the low number of surfaces positive for *C. difficile*, a statistical model could not be constructed for *C. difficile*. Consequently, only descriptive statistics for *C. difficile* are reported.

From the three participating hospitals, 208 different surfaces, for a total of 612 samples, were tested for MRSA, while 191 different surfaces, for a total of 552 samples, were tested for *C. difficile*. Overall, 11.8% (72/612; 95% CI 9.30-14.6%) and 2.4% (13/552; 95% CI 1.3-3.9%) of surfaces sampled were positive for MRSA and *C. difficile*, respectively. For common surfaces, there was no significant difference in the prevalence between MRSA and *C. difficile* (OR=1.13; 95% CI 0.15–6.92; P=0.999). The Spearman’s correlation coefficient indicated that MRSA and *C. difficile* contamination was not correlated at the sample level (ρ=0.05; P=0.233). The proportion of surfaces that tested positive at least once for MRSA or *C. difficile* is presented in Table 2, with Hospital C identified as having the highest prevalence of both MRSA and *C. difficile* in the general environment. Data pertaining to the prevalence of MRSA and *C. difficile* based on visit, ward, surface material, surface location, and surface sampled are presented in Table 3. Over the study period, the prevalence of MRSA and *C. difficile* fluctuated in all three hospitals. None of the negative control cloths tested positive for MRSA or *C. difficile*.

**Table 2:** **Prevalence of MRSA and *C. difficile *contamination of surfaces that tested positive at least once**

**Pathogen**	**Hospital**			**Overall total prevalence (95% CI; n)**
	**A**	**B**	**C**	
	Prevalence (95% CI; n)	Prevalence (95% CI; n)	Prevalence (95% CI; n)	
MRSA	20.5% (12.6-30.4; 18/88)	30.8% (19.9-43.4; 20/65)	47.3% (33.7-61.2; 26/55)	30.8% (24.6-37.5; 64/208)
*Clostridium difficile*	7.3% (2.7-15.2; 6/82)	3.4% (0.4-11.7; 2/59)	10% (3.3-21.4; 5/50)	6.8% (3.7-11.4; 13/191)

CI: confidence interval.

n: number of samples.

**Table 3:** **Descriptive statistics of variables for MRSA and *C. difficile *contamination in the general hospital environment**

	**Hospital**					
	**A**		**B**		**C**	
Variables	MRSA (n)	*C. difficile* (n)	MRSA (n)	*C. difficile* (n)	MRSA (n)	*C. difficile* (n)
Visit:						
1	6.5% (3/46)	2.5% (1/40)	6.7% (3/45)	0% (0/39)	0% (0/42)	0% (0/37)
2	3.7% (2/54)	0% (0/50)	9.1% (5/55)	2.0% (1/49)	53.1% (26/49)	0% (0/44)
3	8.5% (4/47)	0% (0/44)	22.8% (13/57)	2.0% (1/51)	0% (0/51)	2.2% (1/46)
4	18.3% (11/60)	8.8% (5/57)	7.0% (4/57)	0% (0/51)	2.0% (1/49)	9.1% (4/44)
Ward:						
Medical	8.2% (11/135)	1.6% (2/126)	9.2% (6/65)	0% (0/57)	14.8% (17/115)	3.9% (4/103)
Surgical	12.5% (9/72)	6.2% (4/65)	12.8% (19/149)	1.5% (2/133)	13.2% (10/76)	1.5% (1/68)
Surface material:						
Air	6.3% (1/16)	na	12.5% (3/24)	na	0% (0/20)	na
Fabric	8.6% (3/35)	2.9% (1/35)	21.1% (8/38)	2.6% (1/38)	22.4% (15/67)	3.0% (2/67)
Laminate	5.3% (1/19)	0% (0/19)	16.7% (2/12)	0% (0/12)	25.0% (3/12)	16.7% (2/12)
Leather	na	na	na	na	0% (0/2)	0% (0/2)
Metal	9.1% (2/22)	0% (0/22)	14.8% (4/27)	0% (0/27)	10.7% (3/28)	0% (0/28)
Mixed	11.1% (1/9)	0% (0/9)	30.0% (3/10)	10.0% (1/10)	na	na
Plastic	10.7% (11/103)	4.9% (5/103)	5.2% (5/97)	0% (0/97)	11.1% (6/54)	1.9% (1/54)
Wood	33.3% (1/3)	0% (0/3)	0% (0/6)	0% (0/6)	0% (0/8)	0% (0/8)
Surface location:						
Public access	11.0% (17/155)	2.1% (3/144)	11.9% (19/160)	1.4% (2/148)	12.9% (17/131)	3.3% (4/120)
Staff access	5.8% (3/52)	6.4% (3/47)	11.1% (6/54)	0% (0/42)	16.7% (10/60)	1.9% (1/52)
Type of surface:						
Antibacterial wipes container	0% (0/6)	0% (0/6)	25.0% (1/4)	0% (0/4)	na	na
Blood pressure machine	6.7% (1/15)	0% (0/15)	0% (0/12)	0% (0/12)	12.5% (1/8)	0% (0/8)
Brochure holder	0% (0/3)	0% (0/3)	na	na	na	na
Bulletin board	0% (0/2)	0% (0/2)	0% (0/2)	0% (0/2)	28.6% (2/7)	0% (0/7)
Chair back	12.5% (1/8)	0% (0/8)	22.2% (4/18)	0% (0/18)	19.4% (6/31)	0% (0/31)
Chart holder	na	na	0% (0/9)	0% (0/9)	na	na
Clip board	na	na	na	na	12.5% (1/8)	0% (0/8)
Computer keyboard	8.0% (2/25)	12.0% (3/25)	6.9% (2/29)	0% (0/29)	0% (0/8)	0% (0/8)
Counter top	0% (0/9)	0% (0/9)	16.7% (2/12)	0% (0/12)	25.0% (2/8))	12.5% (1/8)
Door knob	0% (0/3)	0% (0/3)	na	na	0% (0/6)	0% (0/6)
Drug cart	19.1% (4/21)	4.8% (1/21)	na	na	0% (0/8)	0 % (0/8)
Elevator panel	0% (0/12)	0% (0/12)	0% (0/12)	0% (0/12)	25.0% (2/8)	0% (0/8)
Glove box holder	33.3% (2/6)	16.7% (1/6)	na	na	na	na
Hand rail	18.8% (3/16)	0% (0/16)	33.3% (4/12)	0% (0/12)	0% (0/8)	0% (0/8)
Heating oven handle	na	na	na	na	14.3% (1/7)	0% (0/7)
Isolation cart	10.0% (1/10)	0% (0/10)	25.0% (3/12)	8.3% (1/12)	14.3% (1/7)	14.3% (1/7)
Isolation gown	25.0% (1/4)	0% (0/4)	10.0% (1/10)	0% (0/10)	0% (0/7)	14.3% (1/7)
Lamp shade	na	na	na	na	33.3% (1/3)	0% (0/3)
Lifter handle	na	na	na	na	0% (0/3)	0% (0/3)
Linen	0% (0/17)	0% (0/17)	0% (0/12)	8.3% (1/12)	23.1% (3/13)	0% (0/13)
Patient chart	8.3% (1/12)	0% (0/12)	0% (0/21)	0% (0/21)	12.5% (1/8)	12.5% (1/8)
Sofa	28.6% (2/7)	14.3% (1/7)	57.1% (4/7)	0% (0/7)	27.3% (3/11)	9.1% (1/11)
Sofa pillow	na	na	na	na	33.3% (1/3)	0% (0/3)
Supply cart	na	na	8.3% (1/12)	0% (0/12)	25.0% (1/4)	0% (0/4)
Telephone	6.7% (1/15)	0% (0/15)	0% (0/6)	0% (0/6)	na	na
Urine collection container	na	na	na	na	20.0% (1/5)	0% (0/5)
Elevator area – air	12.5% (1/8)	na	16.7% (2/12)	na	0% (0/8)	na
Nursing station - air	0% (0/5)	na	8.3% (1/12)	na	0% (0/8)	na
Visiting room - air	0% (0/3)	na	na	na	0% (0/4)	na

na = not applicable as these surfaces/areas were not sampled or were not present in the general environment.

Among the 72 MRSA isolates collected from the hospital environment, seven different *spa* types were identified (Table 4). A total of 60 air samples were taken during the study with 6.7% (n=4) positive for MRSA. All MRSA air isolates were *spa* type 2/t002. Overall, 46 MRSA isolates from patients were obtained during the study. Eight different *spa* types were identified (Table 4).

**Table 4:** Typing data for MRSA isolated from the general environment and patients

**Hospital (n)**	**eGenomics *spa *type**^**a**^	**% per hospital (n)**	**Ridom *spa *type**^**b**^	**PVL genes**	**CMRSA**	**USA equivalent**	**Visit number (n)**			
Environment:							1	2	3	4
A (20)	2	50.0% (10)	t002	No	2	100	2	2	2	4
	539	30.0% (6)	t034	No	No assignment	No assignment	0	0	0	6
	24	10.0% (2)	t242	No	2	100	0	0	1	1
	140	5.0% (1)	t954	No	2	100	1	0	0	0
	957	5.0% (1)	t4867	No	No assignment	No assignment	0	0	1	0
B (25)	2	88.0% (22)	t002	No	2	100	3	5	10	4
	539	12.0% (3)	t034	No	No assignment	No assignment	0	0	3	0
C (27)	1	3.7% (1)	t008	Yes	10	300	0	0	0	1
	7	96.3% (26)	t064	No	5	500	0	26	0	0
Patients:										
A (16)^c^	2	75.0% (12)	t002	No	2	100	0	6	3	3
	23	6.3% (1)	t548	No	2	100	0	1	0	0
	24	6.3% (1)	t242	No	2	100	0	0	1	0
	140	6.3% (1)	t954	No	2	100	0	1	0	0
	696	6.3% (1)	t2069	No	No assignment	No assignment	0	1	0	0
B (25)^d^	2	80.0% (20)	t002	No	2	100	6	4	7	3
	1	4.0% (1)	t008	No	5	500	0	0	1	0
	12	4.0% (1)	t062	No	2	100	1	0	0	0
	23	4.0% (1)	t548	No	2	100	0	0	0	1
	24	4.0% (1)	t242	No	2	100	0	0	0	1
	230	4.0% (1)	t010	No	2	100	1	0	0	0
C (5)^e^	2	80.0% (4)	t002	No	2	100	0	0	2	2
	1	20.0% (1)	t008	Yes	10	300	0	0	1	0

PVL: Panton-Valentine leukocidin.

CMRSA: Canadian epidemic methicillin-resistant *Staphylococcus aureus*.

n = number of samples.

^a^*spa* types classified according to eGenomics (http://tools.egenomics.com)^b^*spa* types classified according to the Ridom system (http://www.spaserver.ridom.de).

^c^ MRSA was isolated from the perianal region (62.5%, n=10) and the anterior nares (37.5%, n=6).

^d^ MRSA was isolated from the perianal region (68%, n=17), the anterior nares (12%, n=3), and from wounds (20%, n=5).

^e^ MRSA was isolated from the anterior nares (100%, n=5).

For *C. difficile*, eight different ribotypes were identified among the 13 isolates from the environment including internationally recognized ribotypes 027 (15.4%, n=2) and 078 (7.7%, n=1). Five *C. difficile* isolates, each representing a different ribotype, were collected from patients from Hospital C only. Data pertaining to the characterization of *C. difficile* isolates are presented in Table 5.

**Table 5:** **Typing data for *C. difficile *isolated from the general environment and patients**

**Hospital (n)**	**Ribotype**	**% per hospital (n)**	**Toxinotype**	**Toxin genes**	**Visit number (n)**			
Environment:					1	2	3	4
A (6)	027	16.7% (1)	III	*tcdA, tcdB, cdtA*	0	0	0	1
	078	16.7% (1)	V	*tcdA, tcdB, cdtA*	1	0	0	0
	MOH-AD	16.7% (1)	III	*tcdA, tcdB, cdtA*	0	0	0	1
	MOH-O	16.7% (1)	0	*tcdA, tcdB*	0	0	0	1
	GRH-A	16.7% (1)	Not tested	None	0	0	0	1
	OVC-J	16.7% (1)	Not tested	None	0	0	0	1
B (2)	027	50.0% (1)	III	*tcdA, tcdB, cdtA*	0	0	1	0
	MOH-C	50.0% (1)	IX	*tcdA, tcdB, cdtA*	0	1	0	0
C (5)	MOH-T	80.0% (4)	0	*tcdA, tcdB*	0	0	1	3
	OVC-J	20.0% (1)	Not tested	None	0	0	0	1
Patients:								
C (5)	MOH-AG	20.0% (1)	0	*tcdA, tcdB*	0	0	1	0
	MOH-T	20.0% (1)	0	*tcdA, tcdB*	0	0	1	0
	MOH-V	20.0% (1)	0	*tcdA, tcdB*	0	1	0	0
	MOH-Y	20.0% (1)	III	*tcdA, tcdB, cdtA*	0	0	1	0
	OS-A	20.0% (1)	Not tested	None	0	0	1	0

n = number of samples.

### Surfaces positive on multiple visits

In Hospital A, a drug cart was contaminated with three different *spa* types, 140/t954 (visit 1), 24/t242 (visit 3), and 2/t002 (visit 4), all consistent with the Canadian epidemic MRSA (CMRSA) 2 clone. In Hospital B, a handrail, an isolation cart, and an air sample taken in the elevator area were contaminated with MRSA on two visits each. For the isolation cart and elevator air sample, all MRSA were identified as *spa* type 2/t002. However, for the handrail, on visit two, *spa* type 2/t002 was identified and on visit 4 *spa* type 539/t034 was identified. In Hospital B, a sofa was identified as being contaminated with *spa* type 2/t002 on three different visits. In Hospital C, the back of a chair located in a nursing station was identified as being contaminated with MRSA on more than one visit; *spa* type 7/t064 on visit two and *spa* type 1/t008 on visit 4. No surfaces were identified as being contaminated with *C. difficile* on more than one visit.

Three surfaces were identified as being contaminated with both MRSA and *C. difficile* on the same visit. These surfaces included a glove box holder and a visiting room sofa in Hospital A and an isolation cart in Hospital B. Four surfaces were identified with being contaminated with MRSA and *C. difficile*, on different visits. These surfaces included a drug cart in Hospital A, and a nursing station counter top, an isolation cart, and a patient chart in Hospital C.

### Statistical model

For the initial univariable analysis, the variables surface material, type of surface, hospital, and the presence of *C. difficile* were significant at the 25% level for the presence of MRSA on a surface (Table 6). There was no statistically significant association in the univariable models between type of ward and MRSA contamination, or surface location and MRSA contamination.

**Table 6:** Univariable logistic regression analysis of variables associated with MRSA contamination

**Variable**	**Description**	**OR**	**95% CI**	**P-value**
Hospital	A	Referent		
	B	1.23	0.66-2.30	0.503
	C	1.54	0.83-2.85	0.169
Ward	Medicine	Referent		
	Surgery	1.21	0.74-1.98	0.443
Surface material	Plastic	Referent		
	Air	0.75	0.25-2.27	0.615
	Fabric	2.41	1.31-4.43	0.005
	Laminate	1.71	0.65-4.50	0.277
	Metal	1.38	0.61-3.13	0.446
	Other^a^	1.65	0.58-4.66	0.346
Surface location	Public access	Referent		
	Staff access	0.96	0.55-1.67	0.881
Type of surface	Computer keyboard	Referent		
	Blood pressure machine	0.88	0.15-5.06	0.885
	Chair back	3.47	1.04-11.60	0.044
	Counter top	2.32	0.54-10.02	0.260
	Drug cart	2.32	0.54-10.02	0.260
	Elevator area^b^	1.74	0.36-8.35	0.489
	Elevator panel	0.97	0.17-5.58	0.970
	Hand rail	3.50	0.95-12.93	0.060
	Isolation cart	3.02	0.75-12.23	0.121
	Isolation gown	1.53	0.26-9.00	0.640
	Linen	1.12	0.24-5.26	0.890
	Nursing station^b^	0.60	0.06-5.69	0.660
	Other^c^	1.96	0.58-6.57	0.276
	Patient chart	0.74	0.13-4.26	0.739
	Sofa	8.16	2.22-29.97	0.002
	Supply cart	2.07	0.34-12.47	0.426
	Telephone	0.73	0.08-6.87	0.779
*C. difficile*	Negative	Referent		
	Positive	2.19	0.59-8.16	0.244

^a^ Includes: leather (n=2), mixed (n=19), and wood (n=17).

^b^ Air samples.

^c^ Surfaces include: antibacterial wipes container (n=10), brochure holder (n=3), bulletin board (n=11), chart holder (n=9), clip board (n=8), door knob (n=9), glove box holder (n=6), heating oven handle (n=7), lamp shade (n=3), lifter handle (n=3), sofa pillow (n=3), urine collection container (n=5), visiting room – air (n=3).

**Figure 1:**
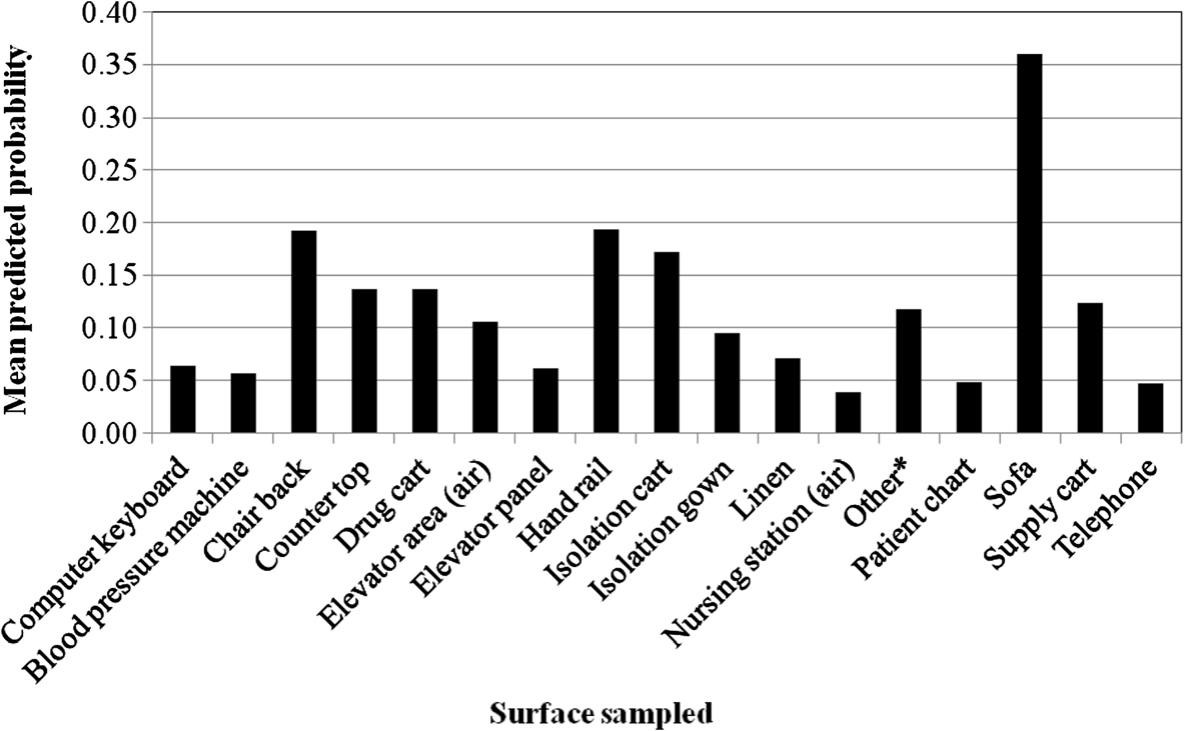
**Mean predicted probability for MRSA to be cultured from surfaces in the general environment.** * Surfaces include: antibacterial wipes container, brochure holder, bulletin board, chart holder, clip board, door knob, glove box holder, heating oven handle, lamp shade, lifter handle, sofa pillow, urine collection container, visiting room – air.

For the final multivariable model, three variables were included: hospital, surface location, and type of surface (Table 7). In constructing the multilevel models for MRSA contamination, the size of the variance components for ward and surface were extremely small (e.g., <10^-6^). Furthermore, the LR test was not statistically significant (P>0.99) comparing models with one or both random intercepts compared to a regular logistic regression model. Similarly, the AIC was smaller when the random effects were not included. Therefore, a regular logistic regression model was used. The variable hospital was forced into the final model as a fixed effect to control for clustering at the hospital level and the potential confounding effects of management differences between hospitals. Surface location was included in the final model as it was a confounder for the variable type of surface. The only statistically significant independent variable in the final model was type of surface. Specifically, results from the multivariable logistic regression model indicated that the odds of contamination for chair backs, hand rails, isolation carts, and sofas were significantly higher than computer keyboards (Table 7). Statistically significant contrasts between the different types of surface categories are presented in Table 8. Similarly, the mean predicted probabilities for surfaces contaminated with MRSA, while fixing hospital and surface location at a referent category, are presented in Figure 1. As demonstrated in both model-based contrasts and the mean predicted probabilities, sofas, hand rails, chair backs, and isolation carts had a higher probability of being contaminated with MRSA compared to other surfaces commonly found in the ward environment. Interactions between variables could not be assessed due to the large number of categories for type of surface and the resulting small number of observations per interaction term.

**Table 7:** Multivariable logistic regression model of variables associated with MRSA contamination

**Variable**	**Description**	**OR**	**95% CI**	**P-value**
Hospital	A	Referent		
	B	1.36	0.69-2.65	0.373
	C	1.19	0.61-2.35	0.606
Surface location	Public access	Referent		
	Staff access	1.99	0.82-4.78	0.126
Type of surface	Computer keyboard	Referent		
	Blood pressure machine	1.58	0.23-10.78	0.638
	Chair back	3.94	1.12-13.86	0.032
	Counter top	3.95	0.76-20.40	0.101
	Drug cart	4.63	0.85-25.17	0.076
	Elevator area^a^	3.03	0.52-17.54	0.216
	Elevator panel	1.72	0.25-11.74	0.580
	Hand rail	6.35	1.38-29.15	0.017
	Isolation cart	5.33	1.07-26.44	0.041
	Isolation gown	2.60	0.37-18.19	0.336
	Linen	2.02	0.35-11.56	0.430
	Nursing station^a^	0.52	0.05-4.92	0.567
	Other^b^	3.24	0.78-13.40	0.105
	Patient chart	0.76	0.13-4.35	0.754
	Sofa	12.92	2.97-56.25	0.001
	Supply cart	2.73	0.42-17.83	0.295
	Telephone	0.87	0.09-8.35	0.904

^a^ Air samples.

^b^ Surfaces include: antibacterial wipes container (n=10), brochure holder (n=3), bulletin board (n=11), chart holder (n=9), clip board (n=8), door knob (n=9), glove box holder (n=6), heating oven handle (n=7), lamp shade (n=3), lifter handle (n=3), sofa pillow (n=3), urine collection container (n=5), visiting room – air (n=3).

**Table 8:** Based on the multivariable logistic regression model, significant model-based contrasts between surfaces contaminated with MRSA

**Surfaces**	**OR**	**95% CI**	**P-value**
Chair back versus Computer keyboard	3.94	1.12-13.86	0.032
Chair back versus Patient chart	5.22	1.06-25.63	0.042
Hand rail versus Computer keyboard	6.35	1.38-29.15	0.017
Hand rail versus Nursing station (air)	12.25	1.18-127.21	0.036
Hand rail versus Patient chart	8.40	1.39-50.97	0.021
Isolation cart versus Computer keyboard	5.33	1.07-26.44	0.041
Isolation cart versus Patient chart	7.05	1.09-45.62	0.040
Sofa versus Blood pressure machine	8.15	1.55-42.81	0.013
Sofa versus Chair back	3.28	1.05-10.24	0.041
Sofa versus Computer keyboard	12.92	2.97-56.25	0.001
Sofa versus Elevator panel	7.51	1.43-39.55	0.017
Sofa versus Linen	6.39	1.51-27.12	0.012
Sofa versus Nursing station (air)	24.91	2.51-247.15	0.006
Sofa versus Other^a^	3.99	1.38-11.52	0.011
Sofa versus Patient chart	17.09	2.95-99.12	0.002
Sofa versus Telephone	14.85	1.55-142.79	0.019

^a^ Surfaces include: antibacterial wipes container (n=10), brochure holder (n=3), bulletin board (n=11), chart holder (n=9), clip board (n=8), door knob (n=9), glove box holder (n=6), heating oven handle (n=7), lamp shade (n=3), lifter handle (n=3), sofa pillow (n=3), urine collection container (n=5), visiting room – air (n=3).

Standardized Pearson residuals were assessed and only two outliers were identified (i.e., residual values of 2.55 and 2.71). For both outliers, data were assessed, found to be recorded correctly, and the observations were kept in the final model. The Pearson χ^2^ test was not significant (P=0.25) indicating that the model fits the data.

## Discussion

This is the first study to explore the epidemiology of both MRSA and *C. difficile* in the general ward environment of community hospitals. By investigating MRSA and *C. difficile* contamination prospectively and under endemic conditions, the information collected can be used by various hospital personnel for surveillance and infection and environmental control measures to reduce the transmission and dissemination of these HA pathogens within the healthcare setting.

Overall, 11.8% and 2.4% of surfaces in the general environment of the medical and surgical wards were contaminated with MRSA and *C. difficile*, respectively. In the literature, the prevalence of MRSA in the hospital environment has ranged from 0.6% to 54% [11,17,35-37] and for *C. difficile* the prevalence has ranged from 2.8% to 50% [14,16,21,38,39]. These wide variations in the reported prevalence of MRSA and *C. difficile* contamination may be attributed to different study designs, including sampling times (endemic versus outbreak settings), the presence of colonized and/or infected patients during sampling, sampling in different hospital wards, sampling pre- and post-disinfection, sampling surfaces once versus multiple times, and the use of different sampling techniques and culture methodologies. Furthermore, the majority of studies investigating MRSA and *C. difficile* have been conducted almost exclusively in patient rooms [12,15,17-22] as opposed to this study which examined the general hospital environment. Therefore, care must be taken when comparing prevalence data between studies.

With respect to the general ward environment, limited information on MRSA and *C. difficile* contamination exists in the literature. Dancer and colleagues [8] screened the computer keyboard, desk, and patient notes located in nursing work stations in two surgical wards during their year-long investigation. In nurses’ work stations, 2.2% (95% CI 0.9-4.6%) of samples were positive for MRSA. Dumford and colleagues [13] conducted a point-prevalence culture survey for toxigenic *C. difficile* strains in physician and nurse work areas, specifically targeting telephones, tabletops, computer keyboards, and door knobs on eight different wards. Results from their investigation revealed that 31% (95% CI 15.3-50.8) and 10% (95% CI 0.3-44.5%) of surfaces in physician and nursing work areas, respectively, were contaminated with *C. difficile*. In the present study, the overall prevalence of MRSA and *C. difficile* on surfaces located in areas accessible only by hospital personnel ranged from 5.8-16.7% and 0-6.4%, respectively. Surfaces sampled included patient charts, computer keyboards, chair backs, and telephones. These surfaces are repeatedly touched by staff throughout the day or immediately after patient contact or leaving an isolation room. Therefore, contamination may be attributed to a number of factors including lack of hand hygiene, failure to use personnel protective equipment, the inability to properly disinfect a surface, inconsistent cleaning schedules, or the quality of cleaning and disinfection protocols.

The dispersal of MRSA in air has been documented in rooms with MRSA positive patients [17,40]. However, information with respect to MRSA outside of patients’ rooms is limited and the role of airborne or aerosolized MRSA in infection or colonization of patients or healthcare workers is not known. In the present study, MRSA was identified in air samples from a nurses’ work station as well as the area located outside the elevator doors. As hospital personnel, patients, and visitors were present in these areas during sampling, it is possible that dust or shed squames containing MRSA from nearby surfaces, the ventilation system, the disposal of linen, or an individual colonized with MRSA may have introduced MRSA in these areas.

In the three participating hospitals, MRSA or *C. difficile* were recovered from unused (clean) linen and/or isolation gowns. Possible reasons for these surfaces being identified with HA pathogens may include contamination from staff, patients, or visitors, cross-contamination from a contaminated storage cart, or contamination during the laundry process. Therefore, it may be necessary to clean and disinfect storage carts to prevent cross-contamination in addition to conducting an audit of the laundry process to ensure that linen and isolation gowns are not contaminated prior to use.

Chair backs, hand rails, isolation carts, and sofas, had an increased odds of being contaminated with MRSA compared to computer keyboards. Model-based contrasts also demonstrated that these four surfaces had increased odds of MRSA contamination compared to other surfaces that were commonly found in the general environment of hospital wards. In addition, Figure 1 illustrates that sofas are especially prone to MRSA contamination. Many of the sampled surfaces were common hand-touch sites not only by hospital personnel but also patients and visitors, thereby increasing the rates of contact along with the number of different people having contact, and subsequently the potential for contamination and transmission. Furthermore, certain surfaces may also be at increased odds for contamination due to the inherent difficulty in cleaning them. Surface material was statistically significant in the univariable analysis, but not in the final model for MRSA contamination. While no significant association was identified, fabric, laminate, and plastic surfaces were found to be contaminated with MRSA and *C. difficile* on multiple occasions and experiments conducted using swatches of fabric and plastic have demonstrated that staphylococci can survive days to months after drying on these types of surfaces [41]. Furthermore, as these surfaces can differ in their texture, they may be more difficult to adequately clean and disinfect. Accordingly, further study of the influence of surface type on the prevalence and persistence of contamination is indicated.

In Canada, epidemic MRSA clones have been designated using pulsed-field gel electrophoresis (PFGE) [26,42]. While PFGE was not performed in this study, a PFGE clone can be inferred from *spa* typing [26]. In Hospital A, *spa* types consistent with CMRSA-2 predominated in both the environment and patients, which is unsurprising since CMRSA-2 is the leading cause of HA-MRSA in Canada [43]. In Hospital B, CMRSA-2 predominated in the environment while both CMRSA-2 and CMRSA-5 were found in patients. CMRSA-5 is an uncommon human epidemic clone typically associated with HA-MRSA infection [43], but is common in people that have contact with horses [44]. Interestingly, in Hospital C, CMRSA-5 was most common in the environment while CMRSA-2 and CMRSA-10 were predominant patient clones. CMRSA-5 was not identified in any patient in Hospital C. The apparent disconnect between patient and environmental MRSA clone distribution in Hospital C is interesting and may suggest unidentified reservoirs or sources, such as hospital staff, visitors, or unscreened patients.

To our knowledge, this is the first report of *spa* type 539/t034 contaminating the hospital environment in Canada. This *spa* type is associated with the livestock-associated (LA) sequence type (ST) 398, although recent evidence indicates it is actually most likely a human *S. aureus* clone that moved into livestock and subsequently acquired methicillin-resistance [45]. This *spa* type is an important cause of community-associated MRSA infection in some regions, particularly northern Europe, most often in individuals with contact with pigs [46-48]. Although this MRSA strain is endemic in the swine population in Canada [49,50] and has also been found in a horse [51] and dogs [52], its role in human infections is unclear. A limited number of human infections have been reported [53], and it remains to be determined whether this is a rare endemic infection, an under diagnosed problem, or an emerging disease in Canada. The relatively high prevalence of environmental contamination with LA-MRSA was surprising given that reports of human infection and colonization in Canada are uncommon and the absence of this strain in patients in this study. The three participating hospitals serve rural communities where pig farming is present, which may increase the likelihood of LA-MRSA exposure. Despite environmental contamination, HA infection with ST398 MRSA was not identified, which is perhaps because this particular clone is known to be relatively inefficiently transmitted in hospitals [54], and is probably less infectious than typical human epidemic clones. Therefore, it is not unreasonable to suspect that *spa* type 539/t034 could be present in colonized patients, healthcare workers, or visitors in the absence of recognized disease in a facility.

In the present study, eight different *C. difficile* ribotypes were identified in the general environment, with six being toxigenic and therefore clinically relevant. However, care must be taken when interpreting the *C. difficile* typing data due to the small number of isolates. Overall, toxinotype 0 (MOH-T and MOH-0) was the most prevalent, but was identified in Hospital C only. The hypervirulent ribotypes 027 and 078 were also identified in the environment of Hospital A and/or B, along with ribotypes MOH-AD and MOH-C. The predominance of toxinotypes 0 and III (ribotype 027) in the environment is not surprising since these were the most common toxinotypes in an earlier study of hospitalized patients in Ontario [55]. However, in that particular study, the highest prevalence of toxinotype 0 strains was associated with ribotype 001, which was not identified in the present investigation.

Currently, there is no universally accepted standard for the surveillance of pathogens from surfaces in the hospital environment [56-58]. A variety of sampling techniques have been employed to recover MRSA and *C. difficile* from the hospital environment including dry or moistened swabs [12,56], sterile gauze pads [13], sterile electrostatic cloths [57], and contact plates [17,56]. Experiments have demonstrated that contact plates had a higher efficiency compared to culture swabs for the recovery of *C. difficile*[59]. However, for MRSA, reports of contact plates [58] and specific types of culture swabs [56] have been identified as efficient and sensitive sampling techniques, respectively. Although contact plates can be processed more quickly than culture swabs [56], contact plates are typically limited to sampling flat surfaces [56,58] whereas culture swabs can be used on irregular surfaces [58]. Like culture swabs, electrostatic cloths can be used to sample irregular surfaces and are also inexpensive, simple to use, and easy to sterilize [57]. In a recent study evaluating *C. difficile* contamination in households, Weese and colleagues [60] isolated *C. difficile* from 5.3% (95% CI 3.8-7.0%) of surfaces using similar sampling and culturing methods as the present investigation.

The present study has several limitations. First, caution should be exercised when interpreting some of the results. For example, although sofas were significantly associated with MRSA contamination, they only constituted seven surfaces in the entire study. Second, not all environmental surfaces were sampled each week. Reasons for surfaces not being sampled four times included equipment/surfaces that had been cleaned by housekeeping the morning of sampling or equipment that was being used by hospital personnel or patients at the time of sampling. This lack of sampling follow-through may bias the overall prevalence of MRSA and *C. difficile* contamination in the hospital environment. Lastly, the discordance of MRSA strains and *C. difficile* ribotypes between the general environment and patient specimens may be attributed to isolates that were not collected and/or patients with MRSA or *C. difficile* that were not identified during the study period.

## Conclusions

The present study demonstrated that MRSA and *C. difficile* were identified from a number of different surfaces in the general ward environment. However, there was no correlation between MRSA and *C. difficile* contamination on these surfaces. As isolation carts, hand rails, chair backs, and sofas were at increased odds of being contaminated with MRSA, protocols are required to ensure that these surfaces are adequately cleaned and disinfected regularly. The identification of LA-MRSA strains in the environment but not patient population in Hospitals A and B requires further surveillance for a better understanding of the epidemiology and microbiology of this emerging MRSA strain. Further studies regarding contact rates among hospital surfaces, type of surface material, and the populations using these surfaces are warranted.

## Abbreviations

MRSA: Methicillin-resistant *Staphylococcus aureus*; LA-MRSA: Livestock-associated methicillin-resistant *Staphylococcus aureus*; CMRSA: Canadian epidemic methicillin-resistant *Staphylococcus aureus*; HA: Hospital-associated; LA: Livestock-associated; PVL: Panton-Valentine leukocidin; *tcdA*: Toxin A; *tcdB*: Toxin B; *cdtA*: Binary toxin; PFGE: Pulsed-field gel electrophoresis; LR: Likelihood ratio; AIC: Akaike’s Information Criteria; ST: Sequence type; CI: Confidence interval; OR: Odds ratio; n: Number of samples.

## Competing interests

The authors declare that they have no competing interests.

## Authors’ contributions

MCF contributed to study design, data collection, analysis, and drafting of the manuscript. DLP and OB contributed to study design and statistical analysis. WAC, KS, and GZ contributed to study design and data collection. JSW contributed to study design and molecular analysis. RRS contributed to study design. All authors contributed to the editing and final version of the manuscript. All authors read and approved the final manuscript.

## Pre-publication history

The pre-publication history for this paper can be accessed here:

http://www.biomedcentral.com/1471-2334/12/290/prepub

## References

[B1] Klevens RM Morrison MA Nadle J Petit S Gershman K Ray S Harrison LH Lynfield R Dumyati G Townes JM Craig AS Zell ER Fosheim GE McDougal LK Carey RB Fridkin SK Active Bacterial Core surveillance (ABCs) MRSA Investigators Invasive methicillin-resistant Staphylococcus aureus infections in the United States J Am Med Assoc 2007 298 1763 1771 10.1001/jama.298.15.1763 17940231

[B2] Gravel D Miller M Simor A Taylor G Gardam M McGeer A Hutchinson J Moore D Kelly S Boyd D Mulvey M Canadian Nosocomial Infection Surveillance Program Health care-associated Clostridium difficile infection in adults admitted to acute care hospitals in Canada: A Canadian nosocomial infection surveillance program study Clin Infect Dis 2009 48 568 576 10.1086/596703 19191641

[B3] Embil JM McLeod JA Al-Barrak AM Thompson GM Aoki FY Witwicki EJ Stranc MF Kabani AM Nicoll DR Nicolle LE An outbreak of methicillin resistant Staphylococcus aureus on a burn unit: potential role of contaminated hydrotherapy equipment Burns 2001 27 681 688 10.1016/S0305-4179(01)00045-6 11600247

[B4] Eggertson L C. difficile outbreaks in Gatineau, Sault Ste. Marie Can Med Assoc J 2007 177 334 335 17698816 10.1503/cmaj.070968 PMC1942105

[B5] Goetghebeur M Landry PA Han D Vicente C Methicillin-resistant Staphylococcus aureus: A public health issue with economic consequences Can J Infect Dis Med Microbiol 2007 18 27 34 18923684 10.1155/2007/253947 PMC2542887

[B6] Miller MA Hyland M Ofner-Agostini M Gourdeau M Ishak M Canadian Hospital Epidemiology Committee, Canadian Nosocomial Infection Surveillance Program Morbidity, mortality, and healthcare burden of nosocomial Clostridium difficile – associated diarrhea in Canadian hospitals Infect Control Hosp Epidemiol 2002 23 137 140 10.1086/502023 11918118

[B7] Shaughnessy MK Micielli RL DePestel DD Arndt J Strachan CL Welch KB Chenoweth CE Evaluation of hospital room assignment and acquisition of Clostridium difficile infection Infect Control Hosp Epidemiol 2011 32 201 206 10.1086/658669 21460503

[B8] Dancer SJ White LF Lamb J Girvan EK Robertson C Measuring the effect of enhanced cleaning in a UK hospital: a prospective cross-over study BMC Med 2009 7 28 10.1186/1741-7015-7-28 19505316 PMC2700808

[B9] Boyce JM Havill NL Otter JA McDonald LC Adams NM Cooper T Thompson A Wiggs L Killgore G Tauman A Noble-Wang J Impact of hydrogen peroxide vapour room decontamination on Clostridium difficile environmental contamination and transmission in a healthcare setting Infect Control Hosp Epidemiol 2008 29 723 729 10.1086/589906 18636950

[B10] Dancer SJ Mopping up hospital infection J Hosp Infect 1999 43 85 100 10.1053/jhin.1999.0616 10549308

[B11] Rohr U Kaminski A Wilhelm M Jurzik L Gatermann S Muhr G Colonization of patients and contamination of the patients’ environment by MRSA under conditions of single-room isolation Int J Hyg Enviro 2009 212 209 215 10.1016/j.ijheh.2008.05.003 18667356

[B12] Rampling A Wiseman S Davis L Hyett AP Walbridge AN Payne GC Cornaby AJ Evidence that hospital hygiene is important in the control of methicillin-resistant Staphylococcus aureus J Hosp Infect 2001 49 109 116 10.1053/jhin.2001.1013 11567555

[B13] Dumford DM 3rd Nerandzic MM Eckstein BC Donskey CJ What is on that keyboard? Detecting hidden environmental reservoirs of Clostridium difficile during an outbreak associated with North American pulsed-field gel electrophoresis type 1 strains Am J Infect Control 2009 37 15 19 10.1016/j.ajic.2008.07.009 19171247

[B14] Martirosian G Recovery of Clostridium difficile from hospital environments J Clin Micro 2006 44 1202 1203 10.1128/JCM.44.3.1202-1203.2006 PMC1393149 16517932

[B15] Verity P Wilcox MH Fawley W Parnell P Prospective evaluation of environmental contamination by Clostridium difficile in isolation side rooms J Hosp Infect 2001 49 204 209 10.1053/jhin.2001.1078 11716638

[B16] Malamou-Ladas H O’Farrell S Nash JQ Tabaqchali S Isolation of Clostridium difficile from patients and the environment of hospital wards J Clin Pathol 1983 36 88 92 10.1136/jcp.36.1.88 6822682 PMC498111

[B17] Sexton T Clarke P O’Neill E Dillane T Humphreys H Environmental reservoirs of methicillin-resistant Staphylococcus aureus in isolation rooms: correlation with patient isolates and implications for hospital hygiene J Hosp Infect 2006 62 187 194 10.1016/j.jhin.2005.07.017 16290319

[B18] French GL Otter JA Shannon KP Adams NM Watling D Parks MJ Tackling contamination of the hospital environment by methicillin-resistant Staphylococcus aureus (MRSA): a comparison between conventional terminal cleaning and hydrogen peroxide vapour decontamination J Hosp Infect 2004 57 31 37 10.1016/j.jhin.2004.03.006 15142713

[B19] Mutters R Nonnenmacher C Susin C Albrecht U Kropatsch R Schumacher S Quantitative detection of Clostridium difficile in hospital environmental samples by real-time polymerase chain reaction J Hosp Infect 2009 71 43 48 10.1016/j.jhin.2008.10.021 19041162

[B20] Dubberke ER Reske KA Noble-Wang J Thompson A Killgore G Mayfield J Camins B Woeltje K McDonald JR McDonald LC Fraser VJ Prevalence of Clostridium difficile environmental contamination and strain variability in multiple health care facilities Am J Infect Control 2007 35 315 318 10.1016/j.ajic.2006.12.006 17577478

[B21] Eckstein BC Adams DA Eckstein EC Rao A Sethi AK Yadavalli GK Donskey CJ Reduction of Clostridium difficile and vancomycin-resistant Enterococcus contamination of environmental surfaces after an intervention to improve cleaning methods BMC Infect Dis 2007 7 61 10.1186/1471-2334-7-61 17584935 PMC1906786

[B22] Samore MH Venkataraman L DeGirolami PC Arbeit RD Karchmer AW Clinical and molecular epidemiology of sporadic and clustered cases of nosocomial Clostridium difficile diarrhea Am J Med 1996 100 32 40 10.1016/S0002-9343(96)90008-X 8579084

[B23] Shopsin B Gomez M Montgomery SO Smith DH Waddington M Dodge DE Bost DA Riehman M Naidich S Kreiswirth BN Evaluation of protein A gene polymorphic region DNA sequencing for typing Staphylococcus aureus strains J Clin Microbiol 1999 37 3556 3563 10523551 10.1128/jcm.37.11.3556-3563.1999 PMC85690

[B24] eGenomicshttp://tools.egenomics.com

[B25] Ridom SpaServerhttp://www.spaserver.ridom.de

[B26] Golding GR Campbell JL Spreitzer DJ Veyhl J Surynicz K Simor A Mulvey MR Canadian Nosocomial Infection Surveillance Program A preliminary guideline for the assignment of methicillin-resistant Staphylococcus aureus to a Canadian pulsed-field gel electrophoresis epidemic type using spa typing Can J Infect Dis Med Microbiol 2008 19 273 281 19436507 10.1155/2008/754249 PMC2604773

[B27] Rankin S Roberts S O’Shea K Maloney D Lorenzo M Benson CE Panton valentine leukocidin (PVL) toxin positive MRSA strains isolated from companion animals Vet Microbiol 2005 108 145 148 10.1016/j.vetmic.2005.02.013 15917142

[B28] Kato H Kato N Watanabe K Iwai N Nakamura H Yamamoto T Suzuki K Kim SM Chong Y Wasito EB Identification of toxin A-negative, toxin B-positive Clostridium difficile by PCR J Clin Microbiol 1998 36 2178 2182 9665986 10.1128/jcm.36.8.2178-2182.1998 PMC105000

[B29] Lemee L Dhalluin A Testelin S Mattrat MA Maillard K Lemeland JF Pons JL Multiplex PCR targeting tpi (triose phosphate isomerise), tcdA (toxin A), tcdB (toxin B), genes for toxigenic culture of Clostridium difficile J Clin Microbiol 2004 42 5710 5714 10.1128/JCM.42.12.5710-5714.2004 15583303 PMC535266

[B30] Stubbs S Rupnik M Gibert M Brazier J Duerden B Popoff M Production of actin-specific ADP-ribosyltransferase (binary toxin) by strains of Clostridium difficile FEMS Microbiol Lett 2000 186 307 312 10.1111/j.1574-6968.2000.tb09122.x 10802189

[B31] Bidet P Barbut F Lalande V Burghoffer B Petit JC Development of a new PCR-ribotyping method for Clostridium difficile based on ribosomal RNA gene sequencing FEMS Microbiol Lett 1999 175 261 266 10.1111/j.1574-6968.1999.tb13629.x 10386377

[B32] Rupnik M Avesani V Janc M von Eichel-Streiber C Delmée M A novel toxinotyping scheme and correlation of toxinotypes with serogroups of Clostridium difficile isolates J Clin Microbiol 1998 36 2240 2247 9665999 10.1128/jcm.36.8.2240-2247.1998 PMC105025

[B33] Dohoo IR Martin W Stryhn H Model-building strategies Veterinary Epidemiologic Research 2003 Prince Edward Island: AVC, Inc, Charlottetown 317 334

[B34] Dohoo IR Martin W Stryhn H Confounder bias: analytic control and matching Veterinary Epidemiologic Research 2003 Prince Edward Island: AVC, Inc, Charlottetown 235 271

[B35] Shelly MJ Scanlon TG Ruddy R Hannan MM Murray JG Meticillin-resistant Staphylococcus aureus (MRSA) environmental contamination in a radiology department Clin Rad 2011 66 861 864 10.1016/j.crad.2011.05.002 21676384

[B36] Dancer SJ Importance of the environment in meticillin-resistant Staphylococcus aureus acquisition: the case for hospital cleaning Lancet Infect Dis 2008 8 101 113 10.1016/S1473-3099(07)70241-4 17974481

[B37] Dancer SJ Coyne M Speekenbrink A Samavedam S Kennedy J Wallace PG MRSA acquisition in an intensive care unit Am J Infect Control 2006 34 10 17 10.1016/j.ajic.2005.08.009 16443087

[B38] Pulvirenti JJ Gerding DN Nathan C Hafiz I Mehra T Marsh D Kocka F Rice T Fischer SA Segreti J Weinstein RA Difference in the incidence of Clostridium difficile among patients infected with human immunodeficiency virus admitted to a public and a private hospital Infect Control Hosp Epi 2002 23 641 647 10.1086/501987 12452290

[B39] Kaatz GW Gitlin SD Schaberg DR Wilson KH Kauffman CA Seo SM Fekety R Acquisition of Clostridium difficile from the hospital environment Am J Epidemiol 1988 127 1289 1294 2835900 10.1093/oxfordjournals.aje.a114921

[B40] Gehanno JF Louvel A Nouvellon M Caillard JF Pestel-Caron M Aerial dispersal of methicillin-resistant Staphylococcus aureus in hospital rooms by infected or colonised patients J Hosp Infect 2009 71 256 262 10.1016/j.jhin.2008.11.015 19162372

[B41] Neely AN Maley MP Survival of enterococci and staphylococci on hospital fabrics and plastic J Clin Microbiol 2000 38 724 726 10655374 10.1128/jcm.38.2.724-726.2000 PMC86187

[B42] Simor AE Ofner-Agostini M Bryce E McGeer A Paton S Mulvey MR Canadian Hospital Epidemiology Committee and Canadian Nosocomial Infection Surveillance Program, Health Canada Laboratory characterization of methicillin-resistant Staphylococcus aureus in Canadian hospitals: Results of 5 years of National Surveillance, 1995-1999 J Infect Dis 2002 186 652 660 10.1086/342292 12195352

[B43] Christianson S Golding GR Campbell J Mulvey MR Canadian Nosocomial Infection Surveillance Program Comparative genomics of Canadian epidemic lineages of methicillin-resistant Staphylococcus aureus J Clin Microbiol 2007 45 1904 1911 10.1128/JCM.02500-06 17428941 PMC1933033

[B44] Anderson MEC Lefebvre SL Weese JS Evaluation of prevalence and risk factors for methicillin-resistant Staphylococcus aureus colonization in veterinary personnel attending an international equine veterinary conference Vet Microbiol 2008 129 410 417 10.1016/j.vetmic.2007.11.031 18187274

[B45] Price LB Stegger M Hasman H Aziz M Larsen J Andersen PS Pearson T Waters AE Foster JT Schupp J Gillece J Driebe E Liu CM Springer B Zdovc I Battisti A Franco A Zmudzki J Schwarz S Butaye P Jouy E Pomba C Porrero MC Ruimy R Smith TC Robinson DA Weese JS Arriola CS Yu F Laurent F Keim P Skov R Aarestrup FM Staphylococcus aureus CC398: host adaptation and emergence of methicillin resistance in livestock MBio 2012 3 e00305 e00311 10.1128/mBio.00305-11 22354957 PMC3280451

[B46] van der Mee-Marquet N François P Domelier-Valentin AS Coulomb F Decreux C Hombrock-Allet C Lehiani O Neveu C Ratovohery D Schrenzel J Quentin R Bloodstream Infection Study Group of Réseau des Hygiénistes du Centre (RHC) Emergence of unusual bloodstream infections associated with pig-borne-like Staphylococcus aureus ST398 in France Clin Infect Dis 2011 52 152 153 10.1093/cid/ciq053 21148535

[B47] van Belkum A Melles DC Peeters JK van Leeuwen WB van Duijkeren E Huijsdens XW Spalburg E de Neeling AJ Verbrugh HA Dutch Working Party on Surveillance and Research of MRSA-SOM Methicillin-resistant and –susceptible Staphylococcus aureus sequence type 398 in pigs and humans Emerg Infect Dis 2008 14 479 483 18325267 10.3201/eid1403.0760 PMC2570802

[B48] Bhat M Dumortier C Taylor BS Miller M Vasquez G Yunen J Brudney K Sánchez-E J Rodriguez-Taveras C Rojas R Leon P Lowy FD Staphylococcus aureus ST398, New York City and Dominican Republic Emerg Infect Dis 2009 15 285 287 10.3201/eid1502.080609 19193274 PMC2657615

[B49] Weese JS Rousseau J Deckert A Gow S Reid-Smith RJ Clostridium difficile and methicillin-resistant Staphylococcus aureus shedding by slaughter-age pigs BMC Vet Res 2011 7 41 10.1186/1746-6148-7-41 21791057 PMC3162498

[B50] Khanna T Friendship R Dewey C Weese JS Methicillin-resistant Staphylococcus aureus colonization in pigs and pig farmers Vet Microbiol 2008 128 298 303 10.1016/j.vetmic.2007.10.006 18023542

[B51] Tokateloff N Manning ST Weese JS Campbell J Rothenburger J Stephen C Bastura V Gow SP Reid-Smith R Prevalence of methicillin-resistant Staphylococcus aureus colonization in horses in Saskatchewan, Alberta, and British Columbia Can Vet J 2009 50 1177 1180 20119542 PMC2764514

[B52] Floras A Lawn K Slavic D Golding GR Mulvey MR Weese JS Sequence type 398 meticillin-resistant Staphylococcus aureus infection and colonisation in dogs Vet Rec 2010 166 826 827 10.1136/vr.b4870 20581361

[B53] Golding GR Bryden L Levett PN McDonald RR Wong A Wylie J Graham MR Tyler S Van Domselaar G Simor AE Gravel D Mulvey MR Livestock-associated methicillin-resistant Staphylococcus aureus sequence type 398 in humans, Canada Emerg Infect Dis 2010 16 587 594 10.3201/eid1604.091435 20350371 PMC3321955

[B54] Wassenberg MW Bootsma MC Troelstra A Kluytmans JA Bonten MJ Transmissibility of livestock-associated methicillin-resistant Staphylococcus aureus (ST398) in Dutch hospitals Clin Microbiol Infect 2011 17 316 319 10.1111/j.1469-0691.2010.03260.x 20459436

[B55] Martin H Willey B Low DE Staempfli HR McGeer A Boerlin P Mulvey M Weese JS Characterization of Clostridium difficile strains isolated from patients in Ontario, Canada, from 2004 to 2006 J Clin Microbiol 2008 46 2999 3004 10.1128/JCM.02437-07 18650360 PMC2546775

[B56] Dolan A Bartlett M McEntee B Creamer E Humphreys H Evaluation of different methods to recover methicillin-resistant Staphylococcus aureus from hospital environmental surfaces J Hosp Infect 2011 79 227 230 10.1016/j.jhin.2011.05.011 21742414

[B57] Murphy CP Reid-Smith RJ Boerlin P Weese JS Prescott JF Janecko N Hassard L McEwen SA Escherichia coli and other selected veterinary and zoonotic pathogens isolated from environmental sites in companion animal veterinary hospitals in southern Ontario Can Vet J 2010 51 963 972 21119862 PMC2920170

[B58] Obee P Griffith CJ Cooper RA Bennion NE An evaluation of different methods for the recovery of methicillin-resistant Staphylococcus aureus from environmental surfaces J Hosp Infect 2007 65 35 41 10.1016/j.jhin.2006.09.010 17140698

[B59] Buggy BP Wilson KH Fekety R Comparison of methods for recovery of Clostridium difficile from an environmental surface J Clin Microbiol 1983 18 348 352 6619285 10.1128/jcm.18.2.348-352.1983 PMC270803

[B60] Weese JS Finley R Reid-Smith RR Janecko N Rousseau J Evaluation of Clostridium difficile in dogs and the household environment Epidemiol Infect 2010 138 1100 1104 10.1017/S0950268809991312 19951453

